# Authors' reply: New metrics to monitor progress towards global HIV targets: using the estimated number of undiagnosed HIV-infected individuals as denominator

**DOI:** 10.2807/1560-7917.ES.2016.21.50.30428

**Published:** 2016-12-15

**Authors:** Anastasia Pharris, Chantal Quinten, Teymur Noori, Andrew J Amato-Gauci, Ard van Sighem

**Affiliations:** 1 European Centre for Disease Prevention and Control (ECDC), Stockholm, Sweden; 2 Stichting HIV Monitoring, Amsterdam, the Netherlands

**Keywords:** HIV infection, PLHIV, undiagnosed, modelling, diagnosis

**To the Editor:** We thank Dr Sasse for his interest in the conclusions of our rapid communication, in which we used the European Centre for Disease Prevention and Control (ECDC) HIV Modelling tool to estimate for the number and proportion of persons living with HIV who remain undiagnosed in the European Union/European Economic Area [1]. In response to our discussion of the limitations of monitoring a proportion of those undiagnosed relative to persons diagnosed and living with HIV, because this will naturally decrease over time as the number of those undiagnosed becomes smaller in relation to the growing number of persons diagnosed and living longer with HIV, Dr Sasse proposes a new metric: the yearly diagnosed fraction (YDF), where diagnoses made in a given year are examined in relation to the number of persons undiagnosed [2]. We agree that the YDF is an interesting proposal which has the potential to inform monitoring of HIV testing programme efforts. However, it should be remembered that metrics based on estimates are not always straightforward to monitor over time. Estimates can change slightly from year to year, depending on data sources and assumptions used in the model, and changes in the YDF should be interpreted with this in mind, and including confidence intervals to understand the range of uncertainty in a given year.
